# 786-0 Renal cancer cell line-derived exosomes promote 786-0 cell migration and invasion *in vitro*

**DOI:** 10.3892/ol.2014.1962

**Published:** 2014-03-11

**Authors:** GANG CHEN, YAO ZHANG, XIAOHOU WU

**Affiliations:** Department of Urology, The First Affiliated Hospital of Chongqing Medical University, Yuzhong, Chongqing 400016, P.R. China

**Keywords:** renal cancer, cancer cell derived-exosomes, invasion, migration, chemokine receptor type 4

## Abstract

Emerging evidence indicates that cancer-derived exosomes contribute to angiogenesis, tumor immunology and invasion. However, whether cancer cell-derived exosomes regulate the migration and invasion of the cancer cell itself, and the underlying mechanisms are not well understood. In the present study, exosomes derived from the 786-0 human renal cancer cell line were isolated, purified and 100 μg/ml were co-cultured with 786-0 cells for 24 h. The 786-0 cells were harvested for a cell invasion and migration assay. The expression of chemokine receptor type 4 (CXCR4) and matrix metalloproteinase-9 (MMP-9) in the 786-0 cells was examined by western blot analysis and revealed that the migration and invasion capabilities of the 786-0 cells were increased, however, the cell adhesion abilities were decreased as a result of the 24-h treatment with 786-0-derived exosomes. Furthermore, the expression levels of CXCR4 and MMP-9 in the 786-0 cells were increased. In conclusion, the 786-0 renal cancer cell line-derived exosomes increased migration and invasion, however, they decreased the adhesion ability of the 786-0 cells. The exosomes may have increased the CXCR4 and MMP-9 expression levels in the 786-0 cells. These findings indicated that renal tumor-derived exosomes may contribute to renal cancer development and progression.

## Introduction

Renal cell carcinoma (RCC) is one of the most common types of genitourinary cancer, accounting for ~2–3% of all malignant tumors (1), and with an incidence of ~209,000 new cases and 102,000 mortalities per year worldwide. In Europe in 2008, 88,300 patients were diagnosed with RCC and 39,230 succumbed to the disease (2). With the development of diagnostic technology, an increasing number of patients with RCC are diagnosed at an early stage. However, a considerable number of patients with RCC present with metastasis at the time of diagnosis. Emerging evidence indicates that the development and progression of RCC are closely associated with the tumor microenvironment (3).

Exosomes are 30–100 nm-sized membrane vesicles, which are released into the tumor microenvironment by the majority of tumor cell types (4). Exosomes are currently recognized as important mediators of cell-to-cell communication, which transfer proteins, mRNAs and microRNAs to neighboring or distant cells to modulate immune function, angiogenesis, cell proliferation and tumor cell invasion (5–7). However, whether cancer cell-derived exosomes regulate the migration and invasion of the cancer cell itself and the underlying mechanisms have not yet been investigated.

Our previous study indicated that tumor cell-derived exosomes contributed to proliferation of the tumor cell (8). In the present study, exosomes were isolated from the 786-0 human renal carcinoma cell line and the effects of 786-0 cell-derived exosomes on the migration and invasion of 786-0 cells as well as the underlying mechanisms were investigated *in vitro*. Our findings may provide a novel insight into the mechanisms underlying the development and progression of RCC.

## Materials and methods

### Cell culture

The 786-0 human renal cancer cell line was purchased from the Institute of Cell Research, Chinese Academy of Sciences (Shanghai, China). The cells were cultured in RPMI-1640 medium (Gibco, Shanghai, China) supplemented with 10% (v/v) fetal bovine serum (FBS; Shijiqing Inc, Beijing, China) at 37°C in an atmosphere of 5% (v/v) CO_2_.

### Isolation and purification of exosomes

The 786-0 cell-derived exosomes were isolated and purified as previously described (9). Briefly, cultured supernatants (100 ml) were harvested and sequentially centrifuged (4°C) at 300, 800 and 10,000 × g for 10, 30 and 30 min, respectively, to remove the pellet and cell debris. The clarified supernatant was then centrifuged at 1,000 × g for 30 min in a 100 kDa MWCO Centrifugal Filter Device (Amicon Drive Systems, Inc., Pineville, NC, USA). The ultracentrifuge supernatant was underplayed with 30% sucrose/D2O density cushion, followed by ultracentrifugation at 100,000 × g for 60 min. The cushion was collected and diluted in phosphate-buffered saline (PBS). The exosomes were further concentrated by centrifuging at 1,000 × g in a 100 kDa MWCO for 30 min. A membrane filter (0.22 μm) was used and following sterilization the exosomes were stored at −80°C. The Bradford method was used to quantify the total protein concentration of exosomes.

### Identification of exosomes

The morphological characteristics of the exosomes were identified under a transmission electron microscope (TEM; JEM-2010, JEOL, Ltd., Tokyo, Japan). The exosomes (20 μl) were resuspended, loaded onto electron microscopy grids and stained with 2% phosphotungstic acid (pH 6.8) for 1 min. The samples were subjected to analysis under TEM at 80 kV. Western blotting was used to identify the molecular composition of G250, intercellular adhesion molecule-1 (ICAM-1) and 70 kDa heat shock protein (Hsp70), which were purchased from Zhongshan Bio-tech Co., Ltd. (Guangdong, China).

### Wound healing assay

The 786-0 cells were divided into three groups as follows: Exosomes (Exo), PBS (PBS) and exosomes depression (Exo-D) groups and pretreated with 100 μg/ml exosomes, 100 μl PBS and 7 mmol/l Amiloride, 5-(N,N-Dimethyl)-, hydrochloride, respectively, for 24 h. Each group was cultured in triplicate. The cells (1×10^5^) were seeded in 12-well plates. After the cells formed a confluent monolayer, a 100 μl tip was used to scratch the monolayer. The cultured medium was replaced with fresh complete medium and the cells were incubated at 37°C in an atmosphere of 5% (v/v) CO_2_. The wound healing was analyzed under a microscope and images were captured 24 h following incubation.

### Invasion assay

The cells were divided into three groups and pretreated in triplicate as described above. The cell invasion assay was performed using transwell chambers with 8 μm pore-sized membranes coated with Matrigel (Collaborative Research, Inc., Bedford, MA, USA) and placed in 24-well plates. The 786-0 cells (1.5×10^4^) were harvested and loaded into the upper segment of the chambers. RPMI-1640 medium with 5% FBS was added to the upper segment of the chamber and the lower chamber contained 10% FBS. Following incubation for 24 h, the upper surfaces of the transwell chambers were wiped with cotton swabs, and the invading cells were fixed and stained with crystal violet solution. The invading cell numbers were counted in five randomly selected microscope fields.

### Cell attachment assays

The 786-0 cells were divided into three groups and pretreated in triplicate as described above. The cells were harvested and seeded onto 6-well culture dishes in RPMI-1640 medium with 10% FBS for 3 h. Cell attachment assays were performed, non-adherent cells were removed and adherent cells were washed twice with PBS and fixed in 95% alcohol. The cell numbers were counted in randomly selected high power fields under an inverted light microscope.

### Western blot analysis

The 786-0 cells were pretreated as previously described for the wound healing assay. The cells were harvested and protein was extracted using a Protein Extraction kit according to the manufacturer’s instructions (Active Motif, Carlsbad, CA, USA). The protein concentrations were determined by the Bradford protein assay. Total protein (10 μg) was separated by SDS-PAGE and transferred onto a polyvinylidene difluoride membrane (Millipore, Billerica, MA, USA). The membranes were blocked with 5% skimmed milk in Tris-buffered saline containing 0.1% Tween-20 for 1 h and incubated with primary antibodies (1:200 dilution) against human chemokine receptor type 4 (CXCR4) and matrix metalloproteinase-9 (MMP-9) obtained from Tianjin Saier Biotechnology Co., Ltd. (Tianjin, China) overnight at 4°C. The bound antibodies were detected with goat anti-rabbit IgG (Zhongshan Bio-tech Co., Ltd.) and visualized using enhanced chemiluminescence reagents (Millipore). The relative levels of each target protein to the control, β-actin, were determined by densitometric analysis using Image J software.

### Statistical analysis

Data are expressed as the mean ± standard deviation. The differences between the experimental groups were determined by Student’s t-test using SPSS 19.0 (IBM, Armonk, NY, USA). P<0.05 was considered to indicate a statistically significant difference.

## Results

### Identification of exosomes derived from the 786-0 cells

**Figure 1:**
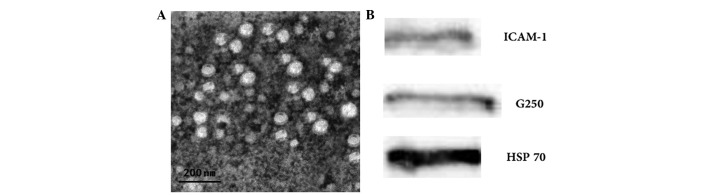
(A) Transmission electron microscopy of exosomes derived from 786-0 cells revealed typical characteristics of cup-shaped or saucer-like structures and 95% ranged between 30 and 100 nm in diameter (magnification, ×200). (B) Western blot analysis of 786-0 cell-derived exosomes revealed the expression of G250, ICAM-1 and HSP70. ICAM-1, intercellular adhesion molecule-1; Hsp70, 70 kDa heat shock protein.

The morphological characteristics of the exosomes derived from 786-0 cells were identified by TEM. The 786-0 cell-derived exosomes exhibited a typical characteristic of a cup-shaped or saucer-like structure and ranged from 30 to 100 nm in diameter (Fig. 1A). Western blot analysis revealed that 786-0 cell-derived exosomes expressed G250, ICAM-1 and Hsp70 (Fig. 1B). The Bradford protein assay found the protein concentration of prepared exosomes was 1,800.7±275.4 μg/ml.

### 786-0 cell-derived exosomes enhance 786-0 cell motility

**Figure 2:**
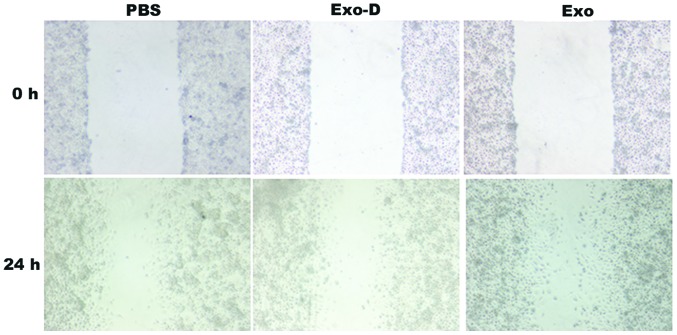
Migration capability of 786-0 cells was analyzed by the wound healing assay. Confluent monolayers of 786-0 cells were scratched and the repair was monitored microscopically after 24 h of pretreatment with Exo (100 μg/ml), PBS (7 mmol) and Amiloride, 5-(N,N-Dimethyl)-, hydrochloride (7 mmol; Exo-D) for 24 h. The width of the wound area narrowed significantly in the Exo-treated group compared with the PBS and Exo-D groups (P<0.05). Exo, exosomes; PBS, phosphate-buffered saline; Exo-D, exosomes depression.

**Figure 3:**
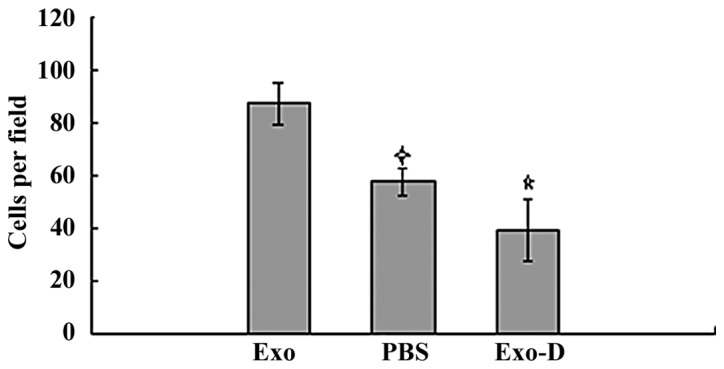
786-0 cell-derived exosomes promote 786-0 cell invasion. 786-0 cells were pretreated with Exo (100 μg/ml), PBS (7 mmol/l) and Amiloride, 5-(N,N-Dimethyl)-, hydrochloride (7 mmol/l; Exo-D) for 24 h. 786-0 cells were seeded in the top chamber of the Matrigel. Following incubation, cells were assessed for invasion as described in the Materials and methods. The number of migrating cells in the Exo-group was significantly increased compared with the PBS and Exo-D groups (P<0.05). Exo, exosomes; PBS, phosphate-buffered saline; Exo-D, exosomes depression. ^*^P<0.05 compared with the Exo group.

Amiloride, 5-(N,N-Dimethyl)-, hydrochloride was reported to depress the cell exosome secretion. In the wound healing assays, the 786-0 cells exhibited an enhanced migratory capacity to the wounded areas in the Exo group compared with the PBS and Exo-D groups (Fig. 2). The Matrigel invasion assays revealed that the 786-0 cells in the Exo group had a higher degree of motility compared with those in the blank control and the Exo-D groups. The invasion ability of cells in the Exo group (87.5±7.8 cells per field) was significantly higher compared with that of cells in the PBS (57.6±5.4 cells per field; P<0.05) and Exo-D (39.3±11.7 cells per field; P<0.05) groups (Fig. 3).

### 786-0 cell-derived exosomes enhance 786-0 cell adhesion

**Figure 4:**
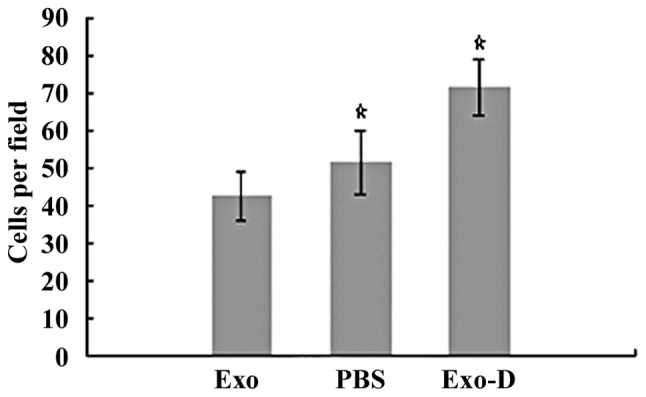
786-0 cell-derived exosomes decrease 786-0 cell attachment. 786-0 cells were pretreated with Exo (100 μg/ml), PBS (7 mmol/l) and Amiloride, 5-(N,N-Dimethyl)-, hydrochloride (7 mmol/l; Exo-D) for 24 h. The cell attachment assays were processed as described in the Materials and methods. Cells in the Exo-D group showed significant enhanced cell attachment to the culture dishes compared with the cells in the Exo and PBS-treated groups (P<0.05). Exo, exosomes; PBS, phosphate-buffered saline; Exo-D, exosomes depression. ^*^P<0.05 compared with Exo-D group.

In the cell attachment assays, cells in the Exo, PBS and Exo-D groups were inoculated in 6-well culture dishes. After the cells were incubated in 10% FBS/ RPMI-1640 medium for 3 h, significantly enhanced cell attachment was observed in the Exo-D group (71.5±7.5 cells per field) compared with attachment in the Exo (42.5±6.5 cells per field; P<0.05) and PBS (51.5±8.5 cells per field; P<0.05) groups (Fig. 4). These findings indicated that 786-0 cell-derived exosomes may decrease the adhesion ability of 786-0 cells.

### 786-0 cell-derived exosomes increase 786-0 cell migration and invasion via the CXCR4 and MMP-9 signaling pathways

**Figure 5:**
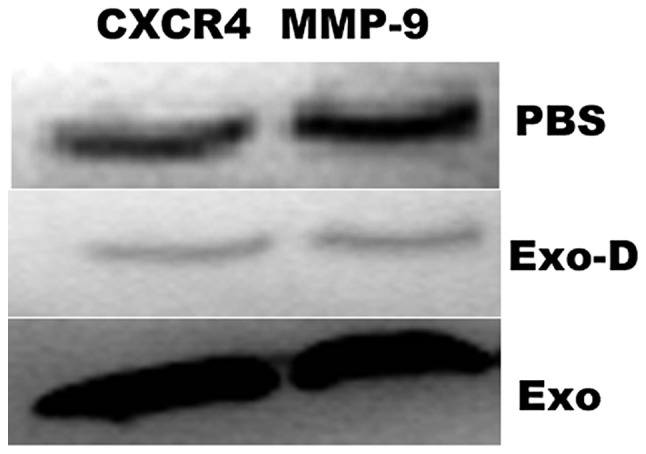
786-0 cell-derived exosomes increase CXCR4 and MMP-9 expression. 786-0 cells were pretreated with Exo (100 μg/ml), PBS (7 mmol/l) and Amiloride, 5-(N,N-Dimethyl)-, hydrochloride (7 mmol/l; Exo-D) for 24 h. Total protein was extracted for western blot analysis. CXCR4 and MMP-9 expression were significantly increased in the Exo-treated group compared with the Exo-D and PBS-treated groups (P<0.05). Exo, exosomes; PBS, phosphate-buffered saline; Exo-D, exosomes depression; CXCR4, chemokine receptor type 4; MMP-9, matrix metalloproteinase-9.

To determine whether exosomes increase 786-0 cell migration and invasion via the CXCR4 and MMP-9 signaling pathways, the protein expression of CXCR4 and MMP-9 was examined by western blott analysis. The protein expression levels of CXCR4 and MMP-9 were identified to be significantly enhanced in the Exo group compared with the PBS group, however, were significantly reduced in the Exo-D group (Exo-D) compared with the PBS group (Fig. 5).

## Discussion

Surgery is the primary curative therapy for patients with local RCC. The treatment for metastatic RCC (mRCC) has been a focus of investigation, however, remains unclear. Analysis of interleukin-2, vascular endothelial growth factor (VEGF) and mammalian target of rapamycin inhibitors have provided improvements in the clinical outcomes of mRCC (10). However, the prognosis for advanced mRCC patients remains poor, with a five-year survival rate of <10% (11). Therefore, further investigations into the underlying mechanisms of mRCC are required.

Tumor-derived exosomes have a bimodal role in cancer progression. First, exosomes regulate the local and systemic environment and contribute to cancer growth and dissemination *in vivo*. Second, exosomes may be important in eliciting the antitumor responses of the immune system (12). Our previous study demonstrated that tumor-derived exosomes enhanced tumor cell proliferation and inhibited Jurkat T-cell proliferation *in vitro* (8). However, whether tumor-derived exosomes enhanced migration and invasion of the cancer cell itself *in vitro,* was not clarified. In the present study, purified exosomes derived from the 786-0 human RCC cell line exhibited typical characteristics, including cup-shaped or saucer-like structures, and ranged between 30 and 100 nm in diameter. Protein analysis revealed that the 786-0 cell-derived exosomes expressed G250, ICAM-1 and Hsp70. In addition, treatment with 100 μg/ml exosomes enhanced cell migration and invasion, but decreased cell attachment *in vitro*. However, the underlying mechanisms by which exosomes enhanced the migration and invasion of 786-0 cells remains unclear.

The invasion of tumor cells is a complex, multistage process, which includes the degradation, by proteases, of the extracellular matrix (ECM) and the basement membrane surrounding the primary tumor, as well as enhancing cell migration and invasion (13,14). Numerous molecules have been associated with cancer metastasis, including MMPs, VEGFs, tumor necrosis factor, platelet-derived growth factor, transforming growth factor-β and Twist-related protein 1 (15). Chemokines and their receptors have been found to contribute to cancer metastasis, particularly stromal cell-derived factor-1α and its receptor CXCR4 (16,17). The CXCR4 chemokine receptor is highly expressed in various types of tumors, including breast, gastric, ovarian, pancreatic, prostate and renal cancers and acute myeloid leukemia (18). Tumors expressing CXCR4 acquire properties that enable them to invade tissue barriers, migrate to secondary organs and form metastases. In patients with RCC, CXCR4 expression has been correlated with a poor overall survival and the expression of CXCR4 and CXCR7 has been considered as a marker, with ~80% accuracy, for predicting RCC metastasis (19,20). As CXCR4 has been associated with the metastasis of renal cancers, CXCR4 expression may be a potential molecular marker for the increased cell invasion and migration abilities, which are induced by tumor-derived exosomes.

MMP-9 is a downstream signaling molecule of CXCR4 and is critical for the migration and invasion of cancer cells (21). MMP-9 belongs to a large family of MMPs, which are responsible for degrading a wide range of ECM components (22). Additionally, MMP-9 is closely associated with tumor invasion and metastasis in a variety of human tumors, including lung adenocarcinoma, hepatocellular carcinoma, and prostate and breast cancers (23–26); furthermore, it was expressed in the serum and urine of patients with RCC (27). Downregulation of forkhead box protein M1 reduced the expression and activity of MMP-2, -9 and VEGF, resulting in the inhibition of migration, invasion and angiogenesis of renal cancer cell lines (28). In the present study, 786-0 cells treated with 100 μg/ml 786-0 cell-derived exosomes showed high migration and invasion capabilities and an increased expression of CXCR4 compared with the PBS-treated group. These findings indicated that the CXCR4 and MMP-9 signaling pathways may be involved in the enhanced migration and invasion capability of 786-0 cells, which was induced by the 786-0 cell-derived exosomes.

In conclusion, these data demonstrated that exosomes, which were derived from the 786-0 human RCC cell lines induced the high migration and invasion capabilities of 786-0 cells *in vitro*. The tumor-derived exosomes induced the tumor cell to highly express CXCR4 and MMP-9. To the best of our knowledge, this is the first study to report that 786-0 cell-derived exosomes enhanced migration and invasion of 786-0 cancer cells. However, further investigations are required to elucidate whether renal tumor-derived exosomes degrade the ECM and promote renal tumor invasion *in vivo.* Our findings may provide novel insights into the tumor progression of RCC.
